# Impact of Left Atrial Ablation on the Atrial Contractile Function: Insights From Intracardiac Echocardiography and Electroanatomical Mapping in Persistent Atrial Fibrillation Ablation

**DOI:** 10.1002/joa3.70179

**Published:** 2025-08-21

**Authors:** Kazuki Noda, Shinichiro Sunamura, Masahiro Suzuki, Kazuyuki Shibutani, Atsushi Tanita, Tsuyoshi Ogata, Toru Takii, Ryoichi Ushigome, Yuji Wakayama, Koji Kumagai, Shigeto Namiuchi

**Affiliations:** ^1^ Department of Cardiology Sendai City Medical Center Sendai Open Hospital Sendai Japan; ^2^ Ushigome Clinic Sendai Japan; ^3^ Wakayama Clinic Sendai Japan; ^4^ Department of Cardiovascular Medicine Tohoku Medical and Pharmaceutical University Sendai Japan

**Keywords:** catheter ablation, intracardiac echocardiography, left atrial contractile function, posterior wall isolation atrial fibrillation

## Abstract

**Background:**

A left atrial (LA) posterior wall isolation (PWI) is a common additional strategy for persistent atrial fibrillation (PeAF) ablation; yet its impact on the LA function remains unclear.

**Objective:**

This study aimed to evaluate the effect of the PWI on the LA contractile function using intracardiac echocardiography (ICE).

**Methods:**

Patients who underwent catheter ablation of PeAF were categorized into extensive encircling pulmonary vein isolation (EEPVI) and PWI groups. The LA contractile function was assessed using the PV reversal wave (PVa) velocity measured by ICE.

**Results:**

No significant difference in the PVa velocity was observed between the EEPVI and PWI group. The EEPVI group patients were categorized into four groups based on scar extent: low‐scar, anterior wall (AW)‐scar‐only, posterior wall (PW)‐scar‐only, and both walls‐scarred. Compared to the low‐scar group, the both walls‐scar group had significantly lower PVa velocities; though no significant difference was found compared to the PW‐scar‐only group. The PVa velocity had a significant correlation with the AW scar. Furthermore, all patients enrolled in this study were also categorized into four groups based on the scar presence, similar to the previous study. Compared with the low‐scar group, the PVa velocities were significantly lower in the AW‐scar‐only and both walls‐scar groups; however, there was no significant difference in the PW‐scar‐only group. The PVa velocity was significantly correlated with the AW scar.

**Conclusion:**

An LA PWI did not significantly impair the LA contractile function. AW scarring appeared to have a greater impact on the LA contractility than the PWI or scarring.

## Graphical abstract

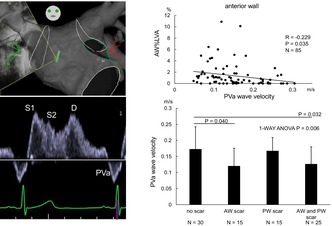

## 1 Introduction

A left atrial (LA) posterior wall, which is composed of tissue primarily derived from a common pulmonary vein, as well as the pulmonary veins, is considered a potential source of atrial fibrillation (AF) triggers and may contribute to AF maintenance [1]. Posterior wall isolation (PWI) involves creating linear lesions between the superior and inferior pulmonary veins bilaterally to electrically isolate the LA posterior wall [2]. Although a PWI has garnered interest as a treatment strategy, large clinical trials have yielded conflicting results regarding its efficacy in preventing AF recurrence. Nevertheless, it remains a frequently chosen option for subsequent ablation procedures [3]. While a radiofrequency‐based PWI can be technically challenging due to the esophageal proximity and catheter maneuverability, cryoablation [4] and pulse field ablation [5] offer simpler approaches, with pulse field ablation providing the added advantage of an esophagus‐independent posterior wall isolation [6]. On the other hand, there is limited data on the extent of the atrial damage caused by these additional ablation techniques, leaving a gap in the evidence needed to make informed risk–benefit assessments when selecting treatment approaches.

Several methods exist for assessing the LA function, including strain [7] and tissue Doppler measurements [8] via transthoracic or transesophageal echocardiography, as well as the LA volume index (LAVI) [9]. The pulmonary vein (PV) flow can be recorded by transthoracic or transesophageal echocardiography, with successful visualization in 73%–95% of adults using transthoracic echocardiography [10]. The atrial systolic pulmonary vein flow serves as an excellent indicator of an active LA contraction, disappearing during AF and being influenced by left ventricular (LV) diastolic function during sinus rhythm. Intracardiac echocardiography (ICE) offers a unique advantage in evaluating the PV flow during pulmonary vein isolation (PVI) procedures. By inserting the ICE catheter into the LA via the transseptal puncture site, it allows for perpendicular imaging of the right superior and inferior pulmonary veins, enabling an accurate assessment of the PV flow. In this study, we utilized ICE to measure the atrial systolic pulmonary vein reversal wave as an indicator of the LA contractile function.

The primary objective of this research was to investigate the impact of an LA posterior wall isolation on the LA contractile function, using the ICE‐derived atrial systolic pulmonary vein flow as a key metric.

## 2 Methods

### 2.1 Study Design and Participants

This single‐center, retrospective observational study was approved by the institutional ethics committee (approval number: 2024‐0097). We included 85 patients (62 males, 23 females) who underwent RF catheter ablation of persistent AF lasting more than 1 week at our institution between July 2022 and December 2024. All patients had transthoracic echocardiography performed during sinus rhythm within 3 months before or after the procedure.

### 2.2 Catheter Ablation Procedure

Catheter ablation was performed under general anesthesia using an iGel airway device, with deep sedation maintained by dexmedetomidine and propofol. The sedation depth was monitored using the Bispectral Index, targeting a range of 40–50 [11]. The LA access was achieved via a transseptal puncture guided by ICE through the left femoral vein.

A PVI was performed using the CARTO system, applying 40 W with a 5–20 g contact force, aiming for an ablation index ≥ 450. Along the esophageal aspect, the power was reduced to 45 W with a target ablation index of 400. Following the PVI, if deemed necessary by the attending physician (e.g., failure to achieve sinus rhythm by cardioversion or extensive posterior wall scarring), a PWI was added using 40 W with a target ablation index of 470–500. Post‐ablation, ICE was used to assess the pulmonary vein flow.

### 2.3 Echocardiographic Assessment

Transthoracic echocardiography was performed within 3 months pre‐ or post‐procedure during sinus rhythm to evaluate the overall cardiac function, including the LV ejection fraction and diastolic function. During the catheter ablation procedure, ICE was used to observe the pulmonary vein flow (Figure S1A,B). As a pilot study, we evaluated pulmonary vein flow in all four PVs in five patients with paroxysmal AF and confirmed that flow patterns were consistent across all PVs within each individual patient (Figure S1C). Thus, we decided to observe the flow of RIPV or RSPV, where the echo beam alignment is most likely to be perpendicular to the flow. Then, PV reversal wave (PVa) flow in patients with paroxysmal AF (*n* = 18) and patients with persistent AF (*n* = 22) was compared before and after treatment (after cardioversion in patients with persistent AF). No significant difference was found between the PVa flow of patients with paroxysmal AF and those with persistent AF after treatment; therefore, it was determined that the PVa in this study can appropriately evaluate LA function (Figure S2). Pulsed‐wave Doppler was employed to measure the peak velocity of the PVa wave.

### 2.4 Posterior Wall Isolation Analysis

We compared the PVa wave velocities measured by ICE between the group that underwent only an extensive encircling pulmonary vein isolation (EEPVI) (EEPVI group) and the group that underwent both an EEPVI and PWI (PWI group). Additionally, we analyzed the patient characteristics and transthoracic echocardiographic parameters between those groups.

### 2.5 Scar Area Analysis

Mapping during a paced rhythm from the right atrium or coronary sinus was performed at the end of the session. Voltage mapping was performed to identify any areas of scar, with bipolar electrogram amplitudes of < 0.5 mV defining areas of scar. We measured the low voltage areas (LVA) on both the anterior and posterior walls of the LA and calculated their percentages relative to the total LA surface area. Those percentages were designated as the ant%LVA for the anterior wall and post%LVA for the posterior wall. To categorize the presence of scar, we calculated the median scar area on the anterior and posterior walls for both the EEPVI group and all patients, including those with a posterior wall isolation. We defined the presence of scar as follows: for the EEPVI group, an ant%LVA ≥ 0.418% and post%LVA ≥ 5.666%; for the entire patient population, including those with a posterior wall isolation, an ant%LVA ≥ 0.674% and post%LVA ≥ 7.374% as thresholds. The patients were categorized into 4 groups based on the extent of scar: low scar on either wall (low scar), anterior wall scar only (AW scar), posterior wall scar only (PW scar), and scars on both walls (AW and PW scar). Analyses were conducted comparing those four groups.

The LA surface area and scar area measurements were performed using a CARTO system. Fast anatomical mapping was created using either the OCTARAY or PENRARAY catheter. The total LA surface area was determined by subtracting the areas of the pulmonary veins, LA appendage, and mitral valve annulus from the overall FAM area. The pulmonary vein boundaries were defined as follows: the anterior wall, roof, and floor were delineated by the pulmonary vein isolation lines on both sides, while the posterior aspect was defined by the intersection of the posterior wall with the plane formed by the anterior wall, roof, and floor lines (Figure S2B).

### 2.6 Statistical Analysis

Comparisons between the two groups were conducted using a Student's *t*‐test for continuous variables and Pearson's chi‐squared test for categorical variables. For multiple group comparisons, a one‐way ANOVA with a Tukey's honestly significant difference test was applied. Continuous variables are presented as the mean ± standard deviation (SD), while categorical variables are shown as numbers and percentages. Statistical significance was set at *p* < 0.05. All analyses were performed using IBM SPSS Statistics, version 21 software (IBM Corp., Armonk, NY, USA).

## 3 Results

### 3.1 Posterior Wall Isolation Analysis

The study cohort was comprised of 24 patients who underwent a posterior wall isolation (PWI) and 61 who did not (EEPVI only). No significant differences were observed in the baseline characteristics such as the age, gender, and BMI between the EEPVI and PWI groups, except for the proportion of hypertension (Table 1).

**TABLE 1:** Baseline characteristics of the patients for the posterior wall isolation analysis.

	EEPVI group	PWI group	*p*
*N*	61/85	24/85	
Age (years)	65.9 ± 10.6	67.4 ± 8.1	0.533
Gender (female)	13/61 (21.3%)	11/24 (45.8%)	0.024
BMI	25.1 ± 4.1	24.6 ± 5.3	0.710
AF duration (months)	25.6 ± 45.5	35.7 ± 38.3	0.376
eGFR (mL/min/1.73 m^2^)	62.0 ± 16.3	59.3 ± 13.5	0.481
NT‐ProBNP (pg/mL)	814.3 ± 918.2	1215.3 ± 1059.2	0.103
CHADs score	1.52 ± 1.20	1.21 ± 0.76	0.242
CHADS‐VASc score	2.26 ± 1.64	2.33 ± 1.21	0.988
CHF	20/61 (32.7%)	11/24 (45.8%)	0.140
HTN	40/61 (65.6%)	9/24 (37.5%)	0.044
DM	11/61 (18.0%)	3/24 (12.5%)	0.536
Prior stroke/TIA	4/61 (6.6%)	0/24 (0%)	0.195
Vascular disease	1/61 (1.6%)	0/24 (0%)	0.528
Age (≥ 75 years)	14/61 (23.0%)	3/24 (12.5%)	0.523
Age (65–74 years)	21/61 (34.4%)	12/24 (50.0%)	0.185

*Note:* The results are expressed as the mean ± SD.

Abbreviations: BMI, body mass index; CHF, congestive heart failure; DM, diabetes mellitus; HTN, hypertension; TIA, transient ischemic attack.

**FIGURE 1:**
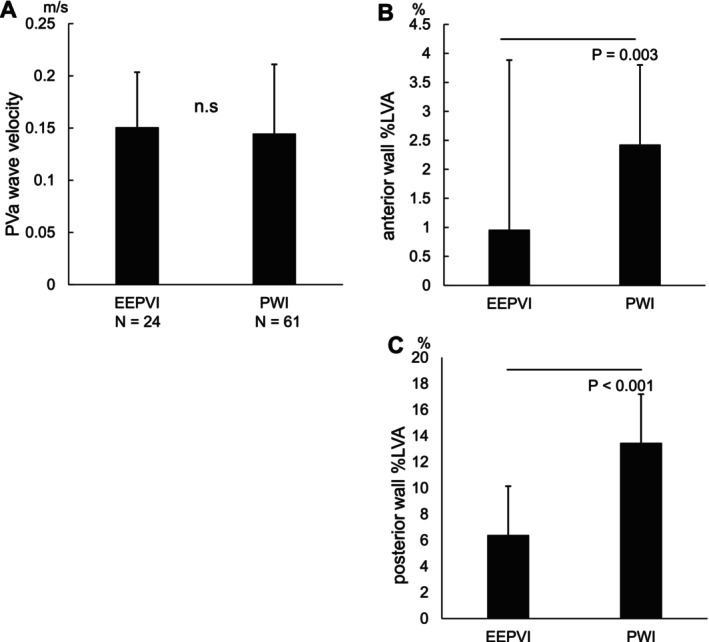
Posterior wall isolation analysis. (A) There was no significant difference in the PVa wave velocity between the EEPVI group (0.151 ± 0.053 m/s) and PWI group (0.145 ± 0.066 m/s). (B) The scar areas on the LA anterior wall were significantly larger in the PWI group than in the EEPVI group (0.96% ± 2.93% vs. 2.43% ± 1.37%, *p* = 0.003). (C) The scar areas on the LA posterior wall were significantly greater in the PWI group than EEPVI group (6.39% ± 3.74% vs. 13.5% ± 3.73%, *p* < 0.001). The data are expressed as the mean SD.

The PWI group exhibited significantly larger scar areas on the LA anterior wall than the EEPVI group (0.96% ± 2.93% vs. 2.43% ± 1.37%, *p* = 0.003). Similarly, the percentage of low‐voltage areas (%LVA) on the LA posterior wall was significantly higher in the PWI group (6.39% ± 3.74% vs. 13.5% ± 3.73%, *p* < 0.001). However, no significant difference was observed in the PVa wave velocity between the EEPVI (0.151 ± 0.053 m/s) and PWI (0.145 ± 0.066 m/s) groups (Figure 1A–C). The echocardiogram parameters including the E/A and E/e', which were indicators of LV diastolic function measured within 3 months after the procedure in sinus rhythm, were not altered between these groups (Table 2).

**TABLE 2:** Echocardiography parameters of the patients in the posterior wall isolation analysis.

	EEPVI group	PWI group	*p*
LVDd (mm)	46.9 ± 5.1	45.8 ± 6.2	0.405
LVDs (mm)	31.6 ± 5.3	32.1 ± 7.6	0.762
LVEF (%)	60.0 ± 8.8	57.8 ± 11.7	0.340
LAD (mm)	39.5 ± 4.6	40.3 ± 8.2	0.622
LAVI (mL/m^2^)	42.9 ± 16.3	49.6 ± 19.6	0.141
E/A	1.23 ± 0.52	1.23 ± 0.37	0.971
E/e'	11.3 ± 3.6	11.2 ± 3.6	0.888

*Note:* The results are expressed as the mean ± SD.

Abbreviations: LAD, left atrial dimension; LAVI, left atrial volume index; LVDd, left ventricular end‐diastolic internal dimension; LVDs, left ventricular end‐systolic internal dimension; LVEF, left ventricular ejection fraction.

### 3.2 Scar Area Analysis in the EEPVI Group

In the EEPVI group, the patients were categorized based on the scar areas on the anterior (AW) and posterior walls (PW). The median values of the scar area percentages (AW: 0.418%, PW: 5.67%) were used to categorize severe myocardial damage and mild myocardial damage. The patients were then divided into four groups: low scar (19 cases), AW scar‐only (12 cases), PW scar‐only (12 cases), and both AW and PW scars (18 cases). No significant differences in the baseline characteristics were observed except for age (65–74 years) among these groups (Table 3).

**TABLE 3:** Baseline characteristics of the patients in the EEPVI group.

	Low scar	AW scar	PW scar	AW + PW scar	*p*
*N*	19/61	12/61	12/61	18/61	
Age (years)	63.6 ± 10.8	68.3 ± 10.4	61.9 ± 9.4	69.2 ± 9.8	0.185
Gender (female)	2/19 (10.5%)	3/12 (25%)	2/12 (16.6%)	6/18 (33.3%)	0.373
BMI	24.0 ± 2.7	24.4 ± 3.3	26.8 ± 4.4	25.4 ± 5.1	0.286
AF duration (months)	35.8 ± 57.4	26.0 ± 60.3	14.4 ± 17.3	22.1 ± 27.9	0.657
eGFR (mL/min/1.73 m^2^)	68.5 ± 15.8	53.9 ± 14.3	65.5 ± 14.2	58.4 ± 16.0	0.058
NT‐ProBNP (pg/mL)	527.8 ± 347.2	854.1 ± 618.6	523.9 ± 358.4	1254.3 ± 1398.3	0.071
CHADs score	1.26 ± 0.85	1.67 ± 1.25	1.58 ± 1.04	1.67 ± 1.49	0.157
CHADS‐VASc score	1.68 ± 1.08	2.41 ± 1.55	2.08 ± 1.50	2.89 ± 2.00	0.253
CHF	3/19 (15.8%)	4/12 (33.3%)	5/12 (41.7%)	8/18 (44.4%)	0.258
HTN	12/19 (63.2%)	8/12 (66.6%)	10/12 (83.3%)	10/18 (55.6%)	0.469
DM	2/19 (10.5%)	2/12 (16.6%)	2/12 (16.6%)	5/18 (27.8%)	0.591
Prior stroke/TIA	2/19 (10.5%)	1/12 (8.3%)	0/12 (0%)	1/18 (5.6%)	0.678
Vascular disease	0/19 (0%)	0/12 (0%)	1/12 (8.3%)	0/18 (0%)	0.246
Age (≥ 75 years)	3/19 (15.8%)	4/12 (33.3%)	2/12 (16.6%)	5/18 (27.8%)	0.618
Age (65–74 years)	4/19 (21.1%)	5/12 (41.7%)	1/12 (8.3%)	11/18 (61.1%)	0.011

*Note:* The results are expressed as the mean ± SD.

Abbreviations: BMI, body mass index; CHF, congestive heart failure; DM, diabetes mellitus; HTN, hypertension; TIA, transient ischemic attack.

**FIGURE 2:**
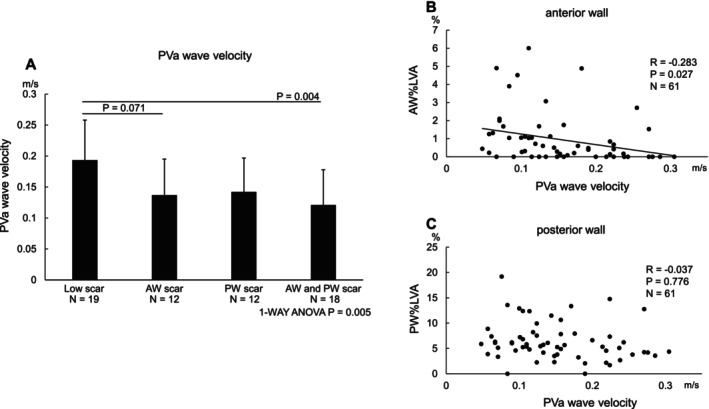
Relation between the LA scar localization and contractile function in the EEPVI only group. (A) The PVa wave had a significantly lower velocity in the both wall scar group (0.121 ± 0.057 m/s, *p* = 0.004) and tended to be lower in the anterior wall scar‐only group (0.137 ± 0.058 m/s, *p* = 0.071) than in the low scar group (0.193 ± 0.064 m/s). No significant difference was observed between the posterior wall scar‐only group (0.142 ± 0.055 m/s) and low scar group. The data are expressed as the mean SD. (B) There was a significant weak negative correlation between the anterior wall %LVA and PVa wave velocity (*r* = −0.283, *p* = 0.027). (C) There was no significant relationship with the posterior wall %LVA (*r* = −0.037, *p* = 0.776).

The PVa wave had a significantly lower velocity in the AW and PW scar groups (0.121 ± 0.057 m/s) as compared to the low scar group (0.193 ± 0.064 m/s), and a trend towards lower values was observed in the AW scar group (0.137 ± 0.058 m/s). However, no significant difference in the PVa wave velocity was observed between the posterior wall scar‐only group (0.142 ± 0.055 m/s) and low scar group (Figure 2A).

Furthermore, the correlation analysis between the PVa wave velocity and %LVA showed no significant relationship with the posterior wall %LVA (*r* = −0.037, *p* = 0.776). However, a weak correlation was observed between the anterior wall %LVA and PVa wave velocity (*r* = −0.283, *p* = 0.027) (Figure 2B,C). The echocardiogram parameters including the E/A and E/e', which were indicators of the LV diastolic function measured within 3 months pre‐ or post‐procedure during sinus rhythm, were not altered in these groups (Table 4).

**TABLE 4:** Echocardiography parameters of the patients in the EEPVI group.

	Low scar	AW scar	PW scar	AW + PW scar	*p*
LVDd (mm)	46.2 ± 5.3	47.0 ± 6.2	49.5 ± 3.8	45.4 ± 3.9	0.051
LVDs (mm)	31.7 ± 5.3	31.1 ± 6.1	33.6 ± 4.3	30.6 ± 5.1	0.418
LVEF (%)	59.9 ± 6.9	62.1 ± 7.7	59.0 ± 9.0	59.4 ± 10.8	0.823
LAD (mm)	39.8 ± 5.2	39.1 ± 3.8	40.9 ± 3.4	38.7 ± 4.7	0.496
LAVI (mL/m^2^)	43.3 ± 13.2	44.2 ± 20.7	44.3 ± 13.0	40.7 ± 17.2	0.939
E/A	1.15 ± 0.39	1.32 ± 0.60	1.17 ± 0.33	1.29 ± 0.64	0.795
E/e'	10.5 ± 3.20	11.8 ± 2.88	11.3 ± 2.81	11.6 ± 4.50	0.753

*Note:* The results are expressed as the mean ± SD.

Abbreviations: LAD, left atrial dimension; LAVI, left atrial volume index; LVDd, left ventricular end‐diastolic internal dimension; LVDs, left ventricular end‐systolic internal dimension; LVEF, left ventricular ejection fraction.

### 3.3 Scar Area Analysis in All Patients

Irrespective of the PWI status, all patients who were enrolled in this study were categorized based on scar areas on the anterior (AW) and posterior walls (PW), and they were divided into four groups: low scar (30 cases), AW scar‐only (15 cases), PW scar‐only (15 cases), and both AW and PW scars (25 cases). The median values of the scar area percentages (AW: 0.674%, PW: 7.374%) were used to categorize severe myocardial damage and mild myocardial damage. No significant differences in the baseline characteristics except for the eGFR and NT‐proBNP were found among these groups (Table 5).

**TABLE 5:** Baseline characteristics of the patients in the scar area analysis.

	Low scar	AW scar	PW scar	AW + PW scar	*p*
*N*	30/85	15/85	15/85	25/85	
Age (years)	63.8 ± 10.3	66.3 ± 12.2	64.2 ± 8.7	70.4 ± 7.2	0.077
Gender (female)	3/30 (10%)	4/15 (26.7%)	6/15 (40%)	11/25 (44%)	0.029
BMI	24.4 ± 3.2	24.6 ± 4.8	27.6 ± 6.8	24.2 ± 3.2	0.100
AF duration (months)	34.9 ± 58.5	8.4 ± 6.3	18.4 ± 21.1	37.7 ± 40.0	0.159
eGFR (mL/min/1.73 m^2^)	67.8 ± 14.7	57.2 ± 15.3	56.7 ± 17.2	58.4 ± 13.2	0.037
NT‐ProBNP (pg/mL)	635.7 ± 478.2	659.6 ± 422.5	1058.8 ± 1326.8	1326.8 ± 1212.6*	0.045
CHADs score	1.27 ± 0.85	1.93 ± 1.06	1.27 ± 0.93	1.44 ± 1.36	0.264
CHADS‐VASc score	1.73 ± 1.15	2.73 ± 1.39	2.20 ± 1.28	2.72 ± 1.89	0.056
CHF	6/30 (20%)	6/15 (40%)	7/15 (46.7%)	13/25 (52%)	0.081
HTN	20/30 (66.7%)	12/15 (80%)	8/15 (53.3%)	10/25 (40%)	0.061
DM	3/30 (10%)	4/15 (26.7%)	3/15 (20%)	4/25 (16%)	0.535
Prior stroke/TIA	2/30 (6.67%)	1/15 (6.7%)	0/15 (0%)	1/25 (4%)	0.755
Vascular disease	0/30 (0%)	0/15 (0%)	1/15 (6.7%)	0/25 (0%)	0.193
Age (≥ 75 years)	5/30 (16.7%)	5/15 (33.3%)	1/15 (6.7%)	7/25 (28%)	0.232
Age (65–74 years)	7/30 (23.3%)	6/15 (40%)	6/15 (40%)	14/25 (56%)	0.104

*Note:* The results are expressed as the mean ± SD.

Abbreviations: BMI, body mass index; CHF, congestive heart failure; DM, diabetes mellitus; HTN, hypertension; TIA, transient ischemic attack.

* *p* = 0.048 versus no scar group.

**FIGURE 3:**
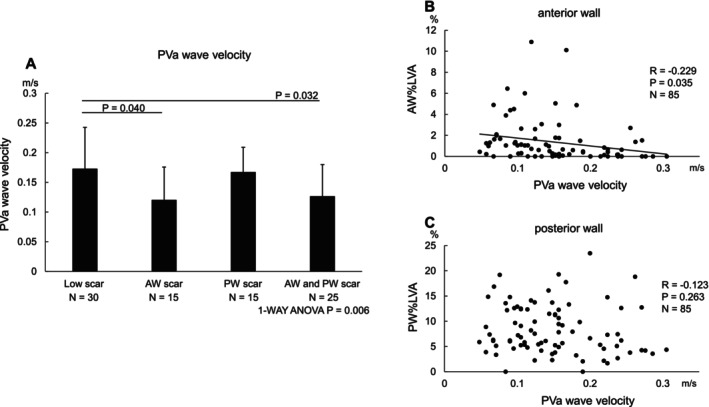
Relation between the LA scar localization and contractile function in the PWI group. (A) The PVa wave had a significantly lower velocity in the anterior wall scar‐only group (0.120 ± 0.055 m/s, *p* = 0.04) and both wall scar group (0.127 ± 0.053 m/s, *p* = 0.032) than in the low scar group (0.173 ± 0.070 m/s). No significant difference was observed between the posterior wall scar‐only group (0.167 ± 0.042 m/s) and low scar group. The data are expressed as the mean SD. (B) There was a significant weak negative correlation between the anterior wall %LVA and PVa wave velocity (*r* = −0.229, *p* = 0.035). (C) There was no significant relationship with the posterior wall %LVA (*r* = −0.123, *p* = 0.263).

The PVa wave analysis clearly revealed significantly lower velocities in the anterior wall scar‐only group (0.120 ± 0.055 m/s) and the both wall scar group (0.127 ± 0.053 m/s) than in the no scar group (0.173 ± 0.070 m/s). However, no significant difference in the PVa wave velocity was observed between the posterior wall scar‐only group (0.167 ± 0.042 m/s) and no scar group (Figure 3A).

The correlation analysis between the PVa wave velocity and %LVA showed no significant relationship with the posterior wall %LVA (*r* = −0.123, *p* = 0.263). However, a weak correlation was observed between the anterior wall %LVA and PVa wave velocity (*r* = −0.229, *p* = 0.035) (Figure 3B,C), similar to the analysis in the EEPVI group. The echocardiogram parameters measured within 3 months pre‐ or post‐procedure during sinus rhythm were not altered in these groups (Table 6).

**TABLE 6:** Echocardiography parameters of the patients in scar area analysis.

	Low scar	AW scar	PW scar	AW + PW scar	*p*
LVDd (mm)	47.2 ± 4.9	45.7 ± 5.8	47.7 ± 5.5	45.6 ± 5.6	0.534
LVDs (mm)	32.7 ± 5.5	30.0 ± 4.9	32.6 ± 6.1	31.2 ± 7.0	0.497
LVEF (%)	58.5 ± 10.2	62.9 ± 6.4	59.1 ± 9.2	58.5 ± 10.7	0.515
LAD (mm)	40.1 ± 4.8	37.4 ± 4.4	40.4 ± 4.4	40.4 ± 7.8	0.417
LAVI (mL/m^2^)	43.6 ± 12.7	40.2 ± 20.9	39.7 ± 9.7	51.2 ± 20.9	0.164
E/A	1.15 ± 0.38	1.22 ± 0.53	1.29 ± 0.29	1.31 ± 0.67	0.677
E/e'	11.0 ± 3.09	11.0 ± 3.76	10.5 ± 2.73	12.3 ± 4.21	0.454

*Note:* The results are expressed as the mean ± SD.

Abbreviations: LAD, left atrial dimension; LAVI, left atrial volume index; LVDd, left ventricular end‐diastolic internal dimension; LVDs, left ventricular end‐systolic internal dimension; LVEF, left ventricular ejection fraction.

## 4 Discussion

This study demonstrated that the LA function was comparable between the groups with and without an LA posterior wall isolation. The PWI group exhibited an increased scar area on both the AW and PW, potentially reflecting more advanced atrial substrate remodeling in the patients selected for this procedure. This observation suggested that patients undergoing a PWI may have more extensive underlying atrial damage. However, it is noteworthy that the PVa flow did not differ significantly between the EEPVI only group and PWI group despite the increased scarring in the former. Further analysis involved stratifying the patients into four groups based on the presence or absence of anterior and posterior scars. That approach revealed that anterior scars were associated with a decrease in the PVa flow. Moreover, a weak significant correlation was observed between the extent of the anterior scar area and PVa flow. Those findings indicated a potential relationship between the location of the atrial scarring and LA function.

In previous studies involving a limited number of cases using MRI to evaluate the LA function in chronic phase [12], it has been reported that a PWI has a minimal impact on the LA function. Similarly, several studies have evaluated LA function using trans‐mitral flow (TMF) as a hemodynamic index following PWI [13, 14]. However, TMF measurements derived from transthoracic echocardiography are recognized to exhibit substantial variability (13%–22%) compared to cardiac MRI, underscoring limitations in reproducibility for quantitative assessments [15]. Furthermore, the PWI is known to have a high rate of reconnections in the chronic phase [16], making it challenging to accurately assess its impact during chronic evaluations. In this study, by confirming that the PWI was complete in all cases and focusing on either the right superior or inferior pulmonary vein due to optimal beam alignment in LA, we were able to accurately evaluate the LA contractile function. Therefore, in this present study, LA contractile function was assessed solely based on acute‐phase PVa velocity measurements, and it remains possible that the findings may not be applicable to chronic‐phase LA function. The mechanism behind the minimal impact of the PWI on the LA contractile function may have been attributed to the thoracic wall providing a foundation for a contraction posteriorly and the fact that the LA posterior wall is electrically the slowest conducting region in the LA, thus inherently contributing less to the LA ejection.

An LA posterior wall isolation is one of the most commonly performed alternative ablation strategies for AF [3]. While current evidence regarding its therapeutic efficacy remains debatable, the lack of reliable methods to identify the AF substrate and its minimal impact on the LA function make a PWI a worthy treatment option for refractory AF. A PWI using radiofrequency ablation often results in incomplete isolation due to catheter manipulation difficulties and esophageal course variations. Moreover, a PWI using radiofrequency ablation rarely causes LA stiff syndrome [17]. However, the increasing adoption of pulse field ablation may allow for simpler and safer PWI procedures [6] without causing LA stiff syndrome [18]. The safety of the PWI regarding the LA function demonstrated in this study may lead to a reconsideration of the PWI indications. In addition, the findings of this study suggested that scarring on the AW of the LA impaired the LA contractile function. None of the patients enrolled in this study underwent anterior wall ablation, and the presence of pre‐existing anterior wall scars was associated with reduced LA contractile function. Therefore, it is considered that the presence of anterior wall scars may contribute to impaired LA contractile function even in the chronic phase. That implies that when treating post‐PVI LA mitral flutter, considering the LA contractile function will be crucial in deciding whether to create an AW or mitral isthmus ablation line, thereby enabling the selection of an appropriate treatment strategy tailored to preserve the LA function.

This study evaluated the LA function using the PVa wave measured by ICE. Although there was no difference in the PVa wave velocity between the EEPVI only group and PWI group, the fact that differences were detected with a certain directionality when dividing the cases into four groups based on the presence or absence of LA scarring on the AW and PW suggests that this method can be considered a reliable indicator for evaluating the LA contractile function. While the LA contraction primarily aims to actively eject blood from the LA to the LV, and the PVa is influenced by the LV diastolic function, this study found no significant differences in the LV diastolic function indices such as the E/A and E/e' among the groups, suggesting that the PVa changes in this study reflected the LA contraction. However, this study did not examine other LA function indices such as the LA strain or tissue doppler, which may be necessary for a more accurate quantification in future studies.

### 4.1 Limitations

This study evaluated the LA function using the PVa wave measured by ICE. However, this study did not examine other LA function indices such as the LA strain or tissue Doppler, which may be necessary for a more accurate quantification in future studies. Additionally, the impact on the overall LA function beyond the contractility remains a topic for future investigation. Moreover, although no apparent signs of acute PV stenosis were observed during measurements, we cannot completely exclude the possibility that edema or other acute post‐ablation and post‐cardioversion changes may have influenced the PVa flow.

## 5 Conclusion

This study demonstrated that an LA posterior wall isolation did not significantly impair the LA contractile function; however, the presence of scar in the LA anterior wall was found to be correlated with a reduction in LA contractility, as measured by intracardiac echocardiography. While the efficacy of the PWI in AF treatment remains debatable, its minimal impact on the LA function positions the PWI as a valuable treatment option for refractory AF.

## Conflicts of Interest

The authors declare no conflicts of interest.

## Supporting information


Data S1:
