# Supplementary material for: Impact of Left Atrial Ablation on the Atrial Contractile Function: Insights From Intracardiac Echocardiography and Electroanatomical Mapping in Persistent Atrial Fibrillation Ablation
Source: J Arrhythm. 2025 Aug 21;41(4):e70179. doi: 10.1002/joa3.70179 (PMC12370844; doi:10.1002/joa3.70179)
Supplement: Supplementary file 1 — Data S1: [file JOA3-41-e70179-s001.zip › joa370179-sup-0001-DataS1/Appendices.docx]

**Supplemental Figure legends**

**Supplemental Figure 1.**

(A) A representative CARTO image showing the positioning of the ICE catheter and the direction of the ultrasound beam used for PV flow assessment. (B) Pulsed-wave Doppler recordings of PV flow in the same patient during AF and after cardioversion to sinus rhythm. The PVa wave is clearly observed only after cardioversion. (C) Comparison of PVa wave velocities recorded from all 4 pulmonary veins (LSPV, LIPV, RSPV, and RIPV) in 5 patients with paroxysmal AF. Each patient exhibited consistent PVa wave velocities across all PVs.

**Supplemental Figure 2.**

(A) Comparison of PVa wave velocity before and after treatment in consecutive 40 patients with AF, including 18 with paroxysmal AF and 22 with persistent AF. In persistent AF patients, post-treatment measurements were taken immediately after cardioversion. No significant difference was observed in post-treatment PVa velocity between the two groups (0.181 ± 0.058 m/s vs 0.176 ± 0.067 m/s). (B) A representative CARTO image showing the low-voltage area (LVA) after extensive encircling pulmonary vein isolation (EEPVI). Ablation extended into the posterior wall of the left atrium, resulting in a clearly delineated LVA (outlined in white).
