# Supplementary material for: Impact of Left Atrial Ablation on the Atrial Contractile Function: Insights From Intracardiac Echocardiography and Electroanatomical Mapping in Persistent Atrial Fibrillation Ablation
Source: J Arrhythm. 2025 Aug 21;41(4):e70179. doi: 10.1002/joa3.70179 (PMC12370844; doi:10.1002/joa3.70179)
Supplement: Supplementary file 1 — Data S1: [file JOA3-41-e70179-s001.zip › joa370179-sup-0001-DataS1/Supplemental Figure 1.pptx]

## Slide 1
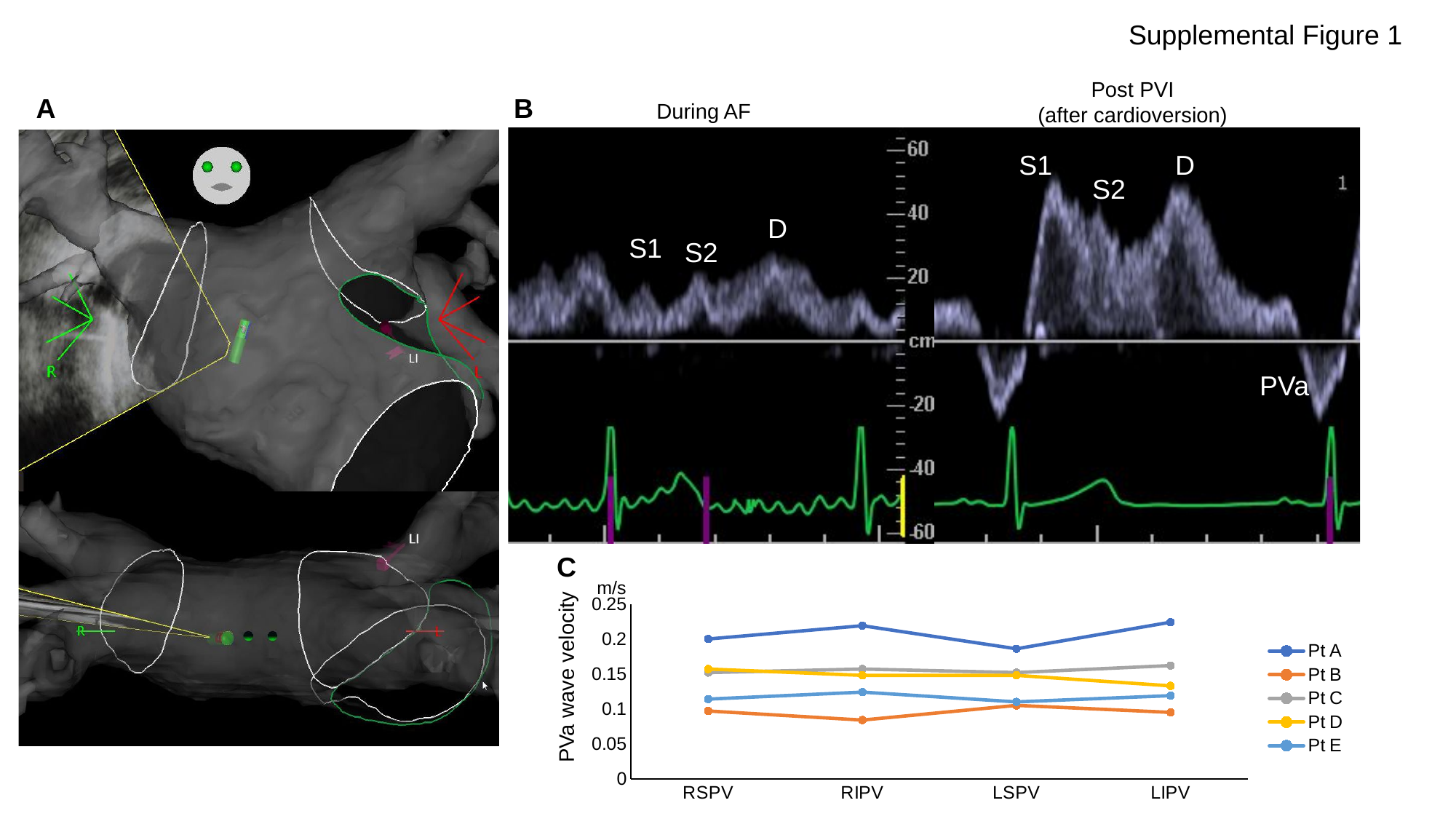

Supplemental Figure 1
Post PVI
(after cardioversion)
A
B
During AF
S1
D
S2
D
S1
S2
PVa
C
m/s
### Chart
| Category | Pt A | Pt B | Pt C | Pt D | Pt E |
|---|---|---|---|---|---|
| RSPV | 0.2 | 0.097 | 0.152 | 0.157 | 0.114 |
| RIPV | 0.219 | 0.084 | 0.157 | 0.148 | 0.124 |
| LSPV | 0.186 | 0.105 | 0.152 | 0.148 | 0.11 |
| LIPV | 0.224 | 0.095 | 0.162 | 0.133 | 0.119 |PVa wave velocity
