# Supplementary material for: Impact of Left Atrial Ablation on the Atrial Contractile Function: Insights From Intracardiac Echocardiography and Electroanatomical Mapping in Persistent Atrial Fibrillation Ablation
Source: J Arrhythm. 2025 Aug 21;41(4):e70179. doi: 10.1002/joa3.70179 (PMC12370844; doi:10.1002/joa3.70179)
Supplement: Supplementary file 1 — Data S1: [file JOA3-41-e70179-s001.zip › joa370179-sup-0001-DataS1/Supplemental Figure 2.pptx]

## Slide 1
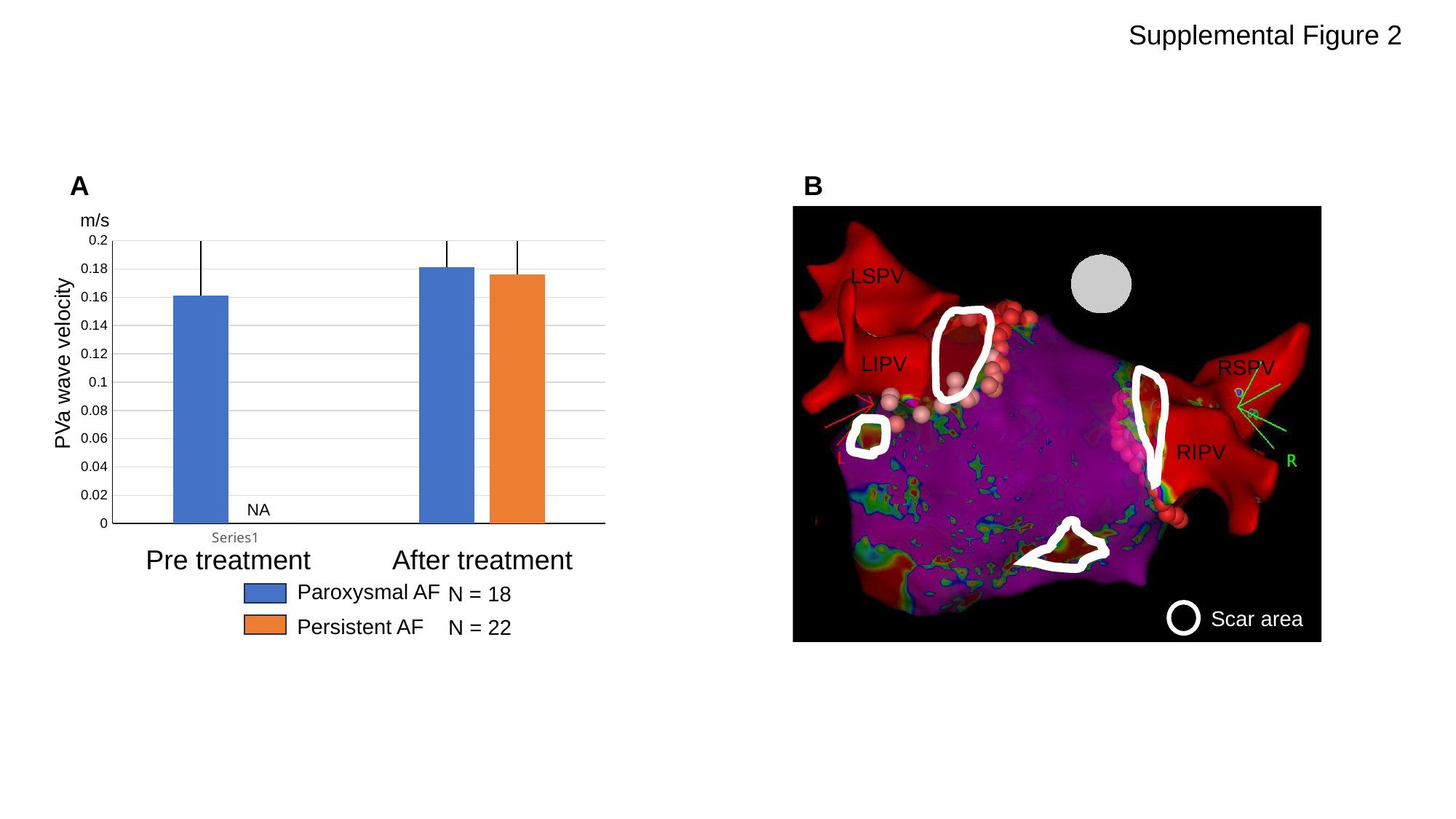

Supplemental Figure 2
A
B
m/s
### Chart
| Category | | |
|---|---|---|
| | 0.1613888888888889 | 0.0 |
| | 0.1813888888888889 | 0.17636363636363636 |LSPV
LIPV
PVa wave velocity
RSPV
RIPV
NA
Pre treatment
After treatment
Paroxysmal AF
N = 18
Scar area
Persistent AF
N = 22
